# A retrospective analysis of determinants of involuntary psychiatric in-patient treatment

**DOI:** 10.1186/s12888-019-2096-5

**Published:** 2019-04-29

**Authors:** Mario Schmitz-Buhl, Stefanie Kristiane Gairing, Christian Rietz, Peter Häussermann, Jürgen Zielasek, Euphrosyne Gouzoulis-Mayfrank

**Affiliations:** 1 LVR Clinics Cologne (LVR-Klinik Köln), Wilhelm-Griesinger-Strasse 23, 51109 Cologne (Köln), Germany; 2 LVR Institute for Healthcare Research, Wilhelm-Griesinger-Strasse 23, 51109 Cologne (Köln), Germany; 3 Current address: St. Agatha Hospital Cologne, Feldgärtenstrasse 97, 50735 Cologne (Köln), Germany; 4 University of Education Heidelberg, Keplerstrasse 87, 69120 Heidelberg, Germany

**Keywords:** Coercion, Involuntary admission, Mental health act, Decision-tree analysis, CHAID

## Abstract

**Background:**

The purpose of our study was to identify predictors of a high risk of involuntary psychiatric in-patient treatment.

**Methods:**

We carried out a detailed analysis of the 1773 mental health records of all the persons treated as in-patients under the PsychKG NRW (Mental Health Act for the state of North Rhine-Westphalia, Germany) in a metropolitan region of Germany (the City of Cologne) in 2011. 3991 mental health records of voluntary in-patients from the same hospitals served as a control group. We extracted medical, sociodemographic and socioeconomic data from these records. Apart from descriptive statistics, we used a prediction model employing chi-squared automatic interaction detection (CHAID).

**Results:**

Among involuntary patients, organic mental disorders (ICD10: F0) and schizophrenia and other psychotic disorders (ICD10: F2) were overrepresented. Patients treated as in-patients against their will were on average older, they were more often retired and had a migratory background. The Exhaustive CHAID analysis confirmed the main diagnosis to be the strongest predictor of involuntary in-patient psychiatric treatment. Other predictors were the absence of outpatient treatment prior to admission, admission outside of regular service hours and migratory background. The highest risk of involuntary treatment was associated with patients with organic mental disorders (ICD 10: F0) who were married or widowed and patients with non-organic psychotic disorders (ICD10: F2) or mental retardation (ICD10: F7) in combination with a migratory background. Also, referrals from general hospitals were frequently encountered.

**Conclusions:**

We identified modifiable risk factors for involuntary psychiatric in-patient treatment. This implies that preventive measures may be feasible and should be implemented to reduce the rate of involuntary psychiatric in-patient treatment. This may include efforts to establish crisis resolution teams to improve out-patient treatment, train general hospital staff in deescalation techniques, and develop special programs for patients with a migratory background.

## Background

The UN convention on the Rights of Persons with Disabilities has raised the public profile of the discussion about the involuntary treatment of people with mental disorders. There is broad societal, ethical and medical consensus that the use of coercive measures including involuntary treatment should be highly restricted [1, 2]. In order to target preventive interventions, it is important to identify risk factors for coercive measures.

On the level of patient-related factors, several clinical, sociodemographic and socioeconomic characteristics may contribute to an increased risk for involuntary psychiatric treatment. In addition, several system factors including the availability and configuration of mental health services, laws and regulations as well as how municipal courts and police services are organized and operate, may modify this risk. Such factors differ largely between countries and regions. Therefore, it is not surprising that detention rates show a marked variability among and within countries [3–6].

One of the most consistent findings from numerous international studies from several European countries and the U.S.A. is that people with a psychotic disorder are at high risk for compulsory admission [3–14]. The association of other mental disorders with a risk for detention has been far less consistent, although there is some indication from studies from Germany, Switzerland, Denmark and the United Kingdom that risk is high for people with bipolar disorder, dementia and other organic psychiatric disorders, and that it is associated with a comorbidity of psychotic and substance use disorders [4, 7, 11, 12, 14]. The high risk for detention among people with certain diagnoses may be mediated by common factors such as symptom severity, imminent danger to others, poor insight and low motivation for treatment [3, 9, 15–17]. Prospective studies from metropolitan regions in the Netherlands and Greece and from India showed that the risk for compulsory treatment was increased by previous involuntary admissions and decreased by regular out-patient contacts in the year before admission [9, 13, 18].

Among sociodemographic factors, male gender and a migratory background were most commonly shown to be associated with involuntary psychiatric treatment in many European countries (United Kingdom, the Netherlands, Ireland, Norway, France, Belgium, Luxembourg, Denmark), the United States and New Zealand [3, 5, 6, 9, 13, 14, 19–23]. However, regarding gender, findings have not been entirely consistent and a small number of studies from Switzerland and some Southern American and Asian countries reported an increased risk for detention in female as compared to male patients [17, 18, 24, 25]. Reports on the role of age have been very inconsistent [9, 11, 12, 14, 15, 18, 23–25]. Among other socioeconomic characteristics on the individual level, a lower level of education, being unmarried, receiving disability pension or social benefits and being unemployed or homeless were identified as risk factors [4, 14, 18, 23, 25]. However, reports have been relatively scarce and some findings are difficult to interpret. For example, in two studies from Amsterdam and Brazil, living with parents and having had longer schooling increased the risk [9, 25], while in a study from Athens, Greece, being married and being divorced/separated decreased the risk for detention [13]. Low social support in the sense of social exclusion on the individual level was found to be a risk factor for involuntary treatment in a retrospective case control study from London [7]. Low perceived social support was also thought to mediate the high detention rates found in populations of immigrants in a recent prospective case study from Greece [13]. On an environmental level, studies from England, Ireland and the Netherlands identified socioeconomic factors such as a high population density in urban regions, high rates of unemployment and aspects of social deprivation in the living area as risk factors for involuntary psychiatric treatment [5, 20, 26].

On the system level of service functioning, studies from England and the Netherlands suggested that lower levels of service integration and longer waiting times for obtaining appropriate mental health care may contribute to higher detention rates [20, 21]. Studies from Norway and Germany showed that presentation in the hospital in the evening, at night and during the weekend was associated with an increased risk for compulsory admission [11, 12, 23]. Finally, regarding laws and regulations, detention rates tended to be lower in countries of the European Union, where the notification or inclusion of a legal representative of the patient in the procedure of detention was mandatory, compared to other EU Member States [3].

Most studies used retrospective designs and analysed existing, routinely collected data from medical case and/or administrative files of one or more hospitals. Few retrospective studies utilized data from official sources such as MHA (Mental Health Act) administrators, registers, national health reports etc. [3, 14, 20, 26]. Some studies used more elaborate prospective designs and analysed data from consecutively admitted cases [8, 9, 13, 15–18, 23, 27]. Overall, prospective studies have several advantages, as they may generate more reliable data and include additional valuable variables such as detailed information on previous history, ratings on symptom severity or insight and self-reports of patients on perceived social support and other relevant aspects. However, most prospective studies either included relatively small study samples ([8], *n* = 227; [15], n=78; [17], *n* = 161; [27], *n*=119; [18], *n* = 300) or they focused on a limited number of possible risk factors for coercion ([13], *n*=946; [23], *n* = 3326). Some large retrospective studies included data from 1000 to nearly 10,000 cases [4, 5, 10, 28] and two studies from Denmark and Germany included data from about 120,000 and 230,000 cases, respectively [12, 14]. However, none of these studies used the full potential of all available clinical data and no study used complex statistical procedures in order to explore possible interactions between different risk factors.

We carried out a thorough retrospective analysis of the full health records of a large sample of psychiatric in-patients. Data were obtained from an in-depth study of all available medical records of all cases. We assessed medical, sociodemographic and socioeconomic data for all cases treated under the Mental Health Act within one year (*n* = 1773) in the German city of Cologne, which has more than one million inhabitants. In addition, we assessed the same data from a larger group of patients who were treated voluntarily in the same hospitals over the same observation period (*n* = 3991). Finally, we employed a modelling approach with a decision-tree-generating algorithm (CHAID: Chi-Square Automatic Interaction Detector [29] with the aim to detect risk factors for involuntary psychiatric admissions and possible interactions between risk factors. The insight derived from this analysis may help to design targeted preventive measures to reduce involuntary psychiatric hospital admissions.

## Methods

### Setting

Cologne is the fourth largest city in Germany with a population of about one million inhabitants (exact population in the year 2011: 1,005,777 inhabitants). It is located in the densely populated and economically strong region of the Rhineland. Apart from a small area in in the northern part of the city, in-patient psychiatric care is provided by four hospitals and it is organized on a sectoral basis: each of the four hospitals provides care to the inhabitants of specific parts of the city ranging from approximately 501,000 to approximately 109,000 inhabitants depending on the size of the hospital. Apart from these four hospitals, there are only few small private psychiatric and psychosomatic units in and near Cologne. These are not responsible for emergency care, have no acute, closed wards and do not treat patients under the Mental Health Act. Out-patient care is mainly provided by a large number of psychiatrists and psychotherapists who work in practices spread across the city. In addition, out-patient units of the psychiatric hospitals provide complex multidisciplinary care to the most severely affected patient groups. In general, the services of the City of Cologne are organized in 9 distinct districts. Community-based social psychiatric services and centres exist in every district. They are coordinated by the Public Health Department and they all work in a similar way with similar resources.

A single Municipal Court is the deciding authority for all detentions in the four psychiatric hospitals of Cologne. The Mental Health Act of the federal state of North Rhine Westphalia (PsychKG NRW) is applicable to individuals who are mentally ill and therefore pose an immediate, severe threat to themselves or others. It is required that there is no other way of averting harm. In most cases, a preliminary detention for up to one day and night is initiated on the basis of a short report of a psychiatrist or a physician with sufficient experience in the field of psychiatry (this includes emergency physicians and staff of emergency units in general hospitals). After this, a court hearing will be held in the hospital on the same day or at the latest on the next day after admission. The court provides a legal representative for the patient. The court will either confirm detention for up to six weeks or it will lift detention immediately. There is no out-patient civil commitment in Germany.

### Study design and data sources

We conducted a retrospective analysis of 5764 health records of inpatients in the four psychiatric hospitals of Cologne (Table 1). We included all patients who were treated under the PsychKG NRW in these hospitals in 2011 (*n* = 1773; including patients who were primarily involuntarily admitted and patients whose status changed from voluntary to involuntary during their in-patient stay in a psychiatric hospital). For feasibility reasons, we were not able to include all voluntary cases of the same year in our analysis (*n* = 8398). We included a large random sample of almost half of the entire voluntary population (*n* = 3991). The random samples of voluntary patients from the three largest hospitals were drawn by using every n^th^ patient based on lists of consecutive voluntary admissions (hospitals 1, 2 and 3, see Table 1). This procedure avoids potential sampling errors due to dates of admission. Regarding the smallest hospital with only 600 cases in 2011, we included all voluntary cases in the analysis.

**Table 1:** Entire population of inpatients in the four psychiatric hospitals of Cologne in the year 2011 and study sample

Entire population					Study sample		
	Voluntary treatment	Involuntary treatment according to PsychKG^a^	Involuntary treatment by guardianship order (BGB)^b^	Total	Voluntary treatment	Involuntary treatment according to PsychKG	Total
Hospital 1	4329	1162	244	5735	2692	1162	3854
Hospital 2	2432	274	151	2857	575	274	849
Hospital 3	1166	222	11	1399	253	222	475
Hospital 4	471	115	14	600	471	115	586
Sum	8398	1773	420	10,591	3991	1773	5764

^a^Mental Health Act: Most patients were admitted under the PsychKG and in the course of treatment they agreed to continue treatment voluntarily. Few patients were admitted voluntarily, but in the course of their stay they were detained under the PsychKG

^b^Treated by guardianship order (BGB – German Civil Code, §§ 1896 ff. BGB)

Five trained assistant physicians extracted the clinical and sociodemographic data from the health records and administrative patient files. These assessors were trained to identify the data of interest in the hospital records and to enter them into a standardized form. We employed standardized training record sets in order to ensure a high degree of care in identifying the data of interest. Diagnoses were routinely assigned by the attending physicians according to the WHO ICD-10 classification. The assessors extracted both the main diagnosis (cause for admissions) and the psychiatric comorbidities (secondary diagnoses). A detailed description of the data set is given in Table 2.

**Table 2:** Data extraction form

Variables	Categories
A. General data	
Case Code	
Postal code (determines hospital in charge)	
Date of admission and discharge	
B. Treatment-related data	
Legal status upon admission/in the course of inpatient treatment	Voluntary/Mental Health Act (PsychKG)/Legal guardianship (BGB)
Hospital where treatment took place	Hospital 1, Hospital 2, Hospital 3, Hospital 4
Time of day of admission	
C. Information on involuntary treatment (PsychKG NRW; Mental Health Act of North Rhine-Westphalia, Germany)	
Time of initiation of PsychKG treatment	Before admission, upon admission, after admission, information not available
Who initiated PsychKG treatment?	Psychosocial service, other hospital, other psychiatric hospital, own hospital, emergency doctor, other/unknown
Reason for PsychKG treatment?	Imminent danger to oneself, to others, both
D. Treatment before admission	
Treatment/contact with professional help system	No previous treatment, psychiatric/psychotherapeutic outpatient treatment, outpatient unit/day hospital, psychosocial services, unknown
Previous inpatient stays at a psychiatric hospital	None, at least one, unknown
Guardianship	Yes – before admission, yes – after admission, no
E. Diagnosis related data	
Main/secondary diagnosis (according to ICD-10, administered by clinician)	F0 (Organic mental disorders), F1 (Substance use disorders), F2 (Psychotic disorders), F3 Affective disorders), F4 (Neurotic disorders), F6 Personality disorders), F7 (Mental retardation), F9 (Behavioural and emotional disorders), other
Suicidal behaviour, self-harm upon admission	Not present, intentional self-harm, suicidal ideations, suicide attempt, unknown
Previous suicide attempts	No, once, several times, unknown
F. Sociodemographic data	
Age	
Gender	Male/female
Marital status	Single, married, widowed, divorced, living apart, unknown
Partnership	Without partner, with partner, unknown
Children	None, one or more children, unknown
Migratory background	No migratory background, probably no migratory background, presumed migratory background, migratory background
Living situation	Home on his own, home together with family/partner, in community, assisted accommodation, emergency accommodation/homeless, unknown
School education	No degree, lower/higher certificate of secondary education, A-levels/high-school diploma, unknown
Professional education	None, apprenticeship, secondary apprenticeship, university degree, unknown
Current professional situation	Employed, unemployed, housewife/−husband, retired, in training, unknown
Degree of employment	None, part time, full time, unknown
Main income source	Employment, pension, own fortune, unemployment benefits, support, unknown

### Statistical analysis

We used χ^2^-tests for the analysis of categorical data (most demographic data, diagnoses and most data on other clinical features and treatment). Due to our sampling procedure, involuntary cases were overrepresented in our samples from hospitals 1–3 (see above, Study design and data sources, and Table 1). Therefore, we weighted the samples from hospitals 1–3 according to the true proportion of voluntary cases in the samples of these hospitals. As a result, the weighted sample matches the true distribution of voluntary and involuntary cases in the four participating hospitals. The analysis of categorical data was performed on the weighted sample (*n* = 10,171). Diagnoses were analysed both on the level of main and secondary diagnoses. In addition, we included the comorbidities addiction and psychosis (F1 and F2) and addiction and personality disorder (F1 and F6) in our analyses, because these were previously shown to be associated with increased risks for aggression, suicide and self-harm, involuntary admissions and containment measures [30–32]. Metric data (age and length of in-patient stay) were analysed by means of ANOVA and t-tests on the unweighted sample (*n* = 5764). The level of significance was set at *p* ≤ 0.01. This conservative significance level was chosen in order to limit the number of potential false positives that could have occured in our analysis otherwise.

As a further means to identify predictor variables for involuntary hospital admissions, we used a statistical modelling approach employing the CHAID algorithm (Chi-Square Automatic Interaction Detector [29, 33]. CHAID is a decision tree-generating algorithm for analysing potentially interacting mixed categorical and continuous data providing a classification of predictive factors of an outcome of interest. Given the multitude of both potentially interacting categorical and continuous potential predictor variables in our study, CHAID was chosen because it provides a data-mining approach for a large dataset as in our study. CHAID is based on the null hypothesis of independence of the predictors and the outcome variable. It performs multiple stepwise χ^2^ tests and provides statistically homogenous subgroups, which are used to generate a summary diagram (“decision tree”) of the relative importance of predictive factors. The decision trees represent both the hierarchy and the interactions of predictive factors for the outcome of interest. We preferred CHAID over multiple binomial logistic regression since decision tree analyses provide a hierarchical risk classification structure which immediately identifies the most promising areas of future preventive interventions. It has been argued that preventive tools based on main effects regression approaches do not adequately reflect the contingent nature of clinical risk assessment processes [34]. CHAID has been used previously in mental healthcare research for example to identify predictors of successful outcomes of methadone treatment [35] and vocational rehabilitation for patients with affective disorders [36]. In our study we carried out an Exhaustive CHAID on the weighted sample (*n* = 10,171). All clinical, treatment-related and sociodemographic items available for both voluntary and involuntary patients (see Table 2, Sections B-F) were included as possible predictor variables in relation to the dichotomous outcome “legal status of hospital treatment” (voluntary vs. involuntary). The model was restricted according to the following stopping criteria: minimum node size to be split *n* = 100, minimum leaf size to be created from a node *n* = 50, or a maximal depth of three. Missing values were treated as valid values, i.e. all cases were used for the computations of both the training and the test model. The model was built with a split-sample validation in a training sample (70%) and then tested with a test sample (30%). The level of significance was set at *p* ≤ 0.05. The results reported here refer to the test sample.

All statistical analyses were carried out using IBM SPSS Statistics Version 25.

## Results

10,591 cases were treated in the four psychiatric hospitals of Cologne during the year 2011. 8398 cases were treated voluntarily, 1773 were detained under the Mental Health Act (PsychKG NRW) and 420 cases were detained by guardianship order (Table 1). The latter were excluded from further analysis. The average quota of involuntary treatment under the PsychKG NRW was 16.7%. The rate of detention under the PsychKG NRW was 1.96 per 1000 inhabitants (1773 detentions per 906.477 inhabitants). The entire clinical population was on average 45.4 years old (SD 16.8), 55.1% were male and 28.2% had a migratory background. The percentages of main diagnoses according to ICD-10 were: F0 (organic mental disorders) 7.4%, F1 (substance use disorders) 34.5%, F2 (schizophrenia and other psychotic disorders) 21.1%, F3 (affective disorders) 27.3%, F4 (neurotic disorders) 5.3%, F6 (personality disorders) 3.2%, F7 (intellectual disabilities) 0.3%, F9 (behavioural and emotional disorders) 0.2%, others 0.8%.

Table 3 summarises all sociodemographic, diagnosis- and treatment-related characteristics of the sample of voluntary and involuntary cases treated under the PsychKG NRW.

**Table 3:** Sociodemographic, diagnosis-related and treatment-related characteristics: groupwise comparisons voluntary vs. involuntary patients

	Category		Voluntary [%[		Involuntary [%]		N	Missings [%]	Statistical measures	P
	Sociodemographic characteristics									
Gender	Female		44.4		45.2		10,171	0	χ^2^(1) = .323	*p* = 0.581
Age (years)	M	SD	44.0	15.1	48.0	20.0	5764	0	T(5762) = −8.484	*p* < 0.001
Marital status	Single		55.3		54.1		9860	3.1	χ^2^(4) = 81.324	p < 0.001
	Married		17.4		20.2					
	Widowed		6.0		10.8					
	Divorced		16.8		11.6					
	Living apart		4.5		3.3					
Relationship	Yes		39.6		38.9		8829	13.2	χ^2^(1) = .235	*p* = 0.644
Children	At least one child		45.2		46.4		9082	10.7	χ^2^(1) = .731	*P* = 0.393
Migration background	Yes		27.7		31.0		10,120	.5	χ^2^(1) = 7.688	*P* = 0.006
Living situation	Home alone		42.3		37.4		9766	4.0	χ^2^(4) = 28.573	*p* < 0.001
	Family/ partner		34.7		36.1					
	Community		3.6		3.6					
	Assisted accomodation		12.2		16.4					
	Emergency accomodation, homeless		7.2		6.4					
School education	No graduation		15.7		17.3		7010	31.1	χ^2^(3) = 3.940	*p* = 0.268
	Lower secondary education		33.4		32.6					
	Higher secondary education		20.8		22.1					
	A-levels/highschool diploma		30.2		27.9					
Professional education	None		38.5		39.6		8117	20.2	χ^2^(3) = 27.309	*p* < 0.001
	Apprenticeship		44.4		38.6					
	Secondary apprenticeship		6.1		9.3					
	University		11.0		12.5					
Current professional situation	Employed		21.2		16.3		8935	12.2	χ^2^(4) = 140.282	*p* < 0.001
	Unemployed		45.1		36.3					
	Housewife/−husband		3.6		5.6					
	Retired		23.8		37.2					
	In training		6.3		4.7					
Degree of employment	None		82.8		88.2		8505	16.4	χ^2^(2) = 25.880	*p* < 0.001
	Full time		13.5		9.2					
	Part time		3.7		2.7					
Main income source	Employment		21.3		17.3		8454	16.9	χ^2^(4) = 112.156	*p* < 0.001
	Pension		25.4		39.0					
	Own fortune		0.8		0.5					
	Unemployment benefits		46.6		36.8					
	Support		5.9		6.4					
Diagnosis-related and treatment-related characteristics										
Main diagnosis ^a^ ICD-10 Codes ^b^	F0		3.9		18.6		10,170	0.0	χ^2^_(6)_ = 845.642	*p* < 0.001
	F1		37.9		23.2					
	F2		17.7		31.4					
	F3		30.8		16.1					
	F4		5.5		5.0					
	F6		3.2		3.7					
	Other		1.0		2.0					
Main or secondary diagnoses ICD-10 Codes ^b^	F0		5.1		20.4		10,171	0	χ^2^_(1)_ = 479.289	*p* < 0.001
	F1		57.8		47.0		10,171	0	χ^2^_(1)_ = 69.080	*p* < 0.001
	F2		20.7		34.9		10,171	0	χ^2^_(1)_ = 163.635	*p* < 0.001
	F3		42.5		22.4		10,171	0	χ^2^_(1)_ = 246.564	*p* < 0.001
	F4		15.4		9.3		10,171	0	χ^2^_(1)_ = 44.513	*p* < 0.001
	F6		15.6		14.0		10,171	0	χ^2^_(1)_ = 2.806	*p* = 0.096
	F7		1.2		1.3		10,171	0	χ^2^_(1)_ = .173	*p* = 0.642
	F9		1.5		0.6		10,171	0	χ^2^_(1)_ = 9.213	*p* = 0.003
Psychiatric comorbidities (dual diagnoses)	F1 + F2		8.4		12.0		10,171	0	χ^2^_(1)_ = 22.647	*p* < 0.001
	F1 + F6		8.6		8.7		10,171		χ^2^_(1)_ = 0.033	*p* = 0.856
Legal guardianship	Yes, before admission		15.3		28.7		8643	15.0	χ^2^_(1)_ = 134.794	*p* < 0.001
Suicidal tendency upon admission	Present		20.9		37.9		10,133	.4	χ^2^_(1)_ = 230.003	*p* < 0.001
Previous suicide attempts	Yes		25.0		30.1		8332	18.1	χ^2^_(1)_ = 14.977	*p* < 0.001
Length of in-patient stay (days)	M	SD	20.7	*25.5*	27.9	*36.8*	5763	0	T(2.556) = − 7.489	*p* < 0.001
Treatment before admission	No previous treatment		37.3		38.9		10,171	0	χ^2^_(1)_ = 1.619	*p* = 0.205
	Out-patient psychiatric/psychotherapeutic treatment		33.1		31.1		10,171	0	χ^2^_(1)_ = 2.603	*p* = 0.113
	Psychosocial service/psychosocial centre		1.0		2.4		10,171	0	χ^2^_(1)_ = 21.306	*p* < 0.001
	Out-patient unit/day hospital		22.5		16.7		10,171	0	χ^2^_(1)_ = 29.072	*p* < 0.001
Time of admission	Regular service hours: 8 a.m. - 4 p.m., Mondays-Fridays		61.1		37.1		10,171	0	χ^2^_(1)_ = 342.021	*p* < 0.001
	Outside of regular service hours		38.9		62.9					
Previous psychiatric hospital stays ^c^	None		22.8		28.4		9848	3.2	χ^2^_(1)_ = 23.240	*p* < 0.001
	At least one		77.2		71.6					

^a^Main diagnosis: Diagnosis that was the cause for admission

^b^ICD-10 Codes: F0 Organic mental disorders, F1 Substance use disorders, F2 Schizophrenia and other psychotic disorders, F3 Affective disorders, F4 Neurotic disorders, F6 Personality disorders, F7 Intellectual disabilities, F9 Behavioural and emotional disorders

^c^At any point in time prior to the current stay

### Clinical variables

Organic mental disorders (ICD10: F0) and psychotic disorders (ICD 10: F2) were overrepresented in the group of involuntary patients (F0: 18.6% vs. 3.9%, F2: 31.4% vs. 17.7%), whereas substance use disorders (ICD 10: F1) and affective disorders (ICD 10: F3) were more common among the group of voluntarily treated patients (F1: 37.9% vs. 23.2%, F3: 30.8% vs. 16.1%). These findings were robust when either the main diagnosis alone or the main and secondary diagnoses were taken into account for the analysis (Table 3). The comorbidity of addiction and psychosis (F1 and F2) was overrepresented among involuntary patients (12.0% vs. 8.4%). Prior to the admission, involuntary patients were more likely to be in touch with community psychosocial services (2.4% vs. 1.0%) and to be under legal guardianship (28.7% vs. 15.3%), and they were less likely to having received psychiatric treatment in an outpatient unit (31.1% vs. 33.1%). Involuntary patients were admitted for hospital treatment more often outside of regular service hours (62.9% vs. 38.9%). Finally, involuntary patients were admitted more often due to suicidal behaviour and self-harm (37.9% vs. 20.9%) and reported more often a history of suicidal attempts compared to voluntary patients (30.1% vs. 25.0%). There was an appreciable percentage of missing values for the items “previous suicide attempts” and “legal guardianship prior to admission” (18.1 and 15%, resp.), otherwise data quality was satisfactory (Table 3).

### Sociodemographic items

Patients treated under the Mental Health Act were older than voluntarily treated patients (48.0 vs. 44.0 years). They were more often retired (37.2% vs. 23.8%) including early retirement due to illness. They more often presented with a migratory background (31.0% vs. 27.7%). They lived more often in supervised accommodation (16.4% vs. 12.2%) and less often on their own (37.4% vs. 42.3%) (Table 3). Patients treated under the Mental Health Act differed from the voluntarily treated patients in terms of their professional training: they had received more often higher levels of education such as a secondary apprenticeship (9.3% vs. 6.1%) or a university degree (12.5% vs. 11.0%). However, they were less often in active employment (16.3% vs. 21.2%) and their main income source was more often a pension (39.0% vs. 25.4%). Despite efforts to extract all necessary data from the available medical records, the percentage of missing values on some sociodemographic data was high (e.g., school education 31.1%, professional education 20.2%, degree of employment 16.4% and main income source 16.9%) (Table 3).

### Decision-tree analysis with CHAID

**Fig. 1:**
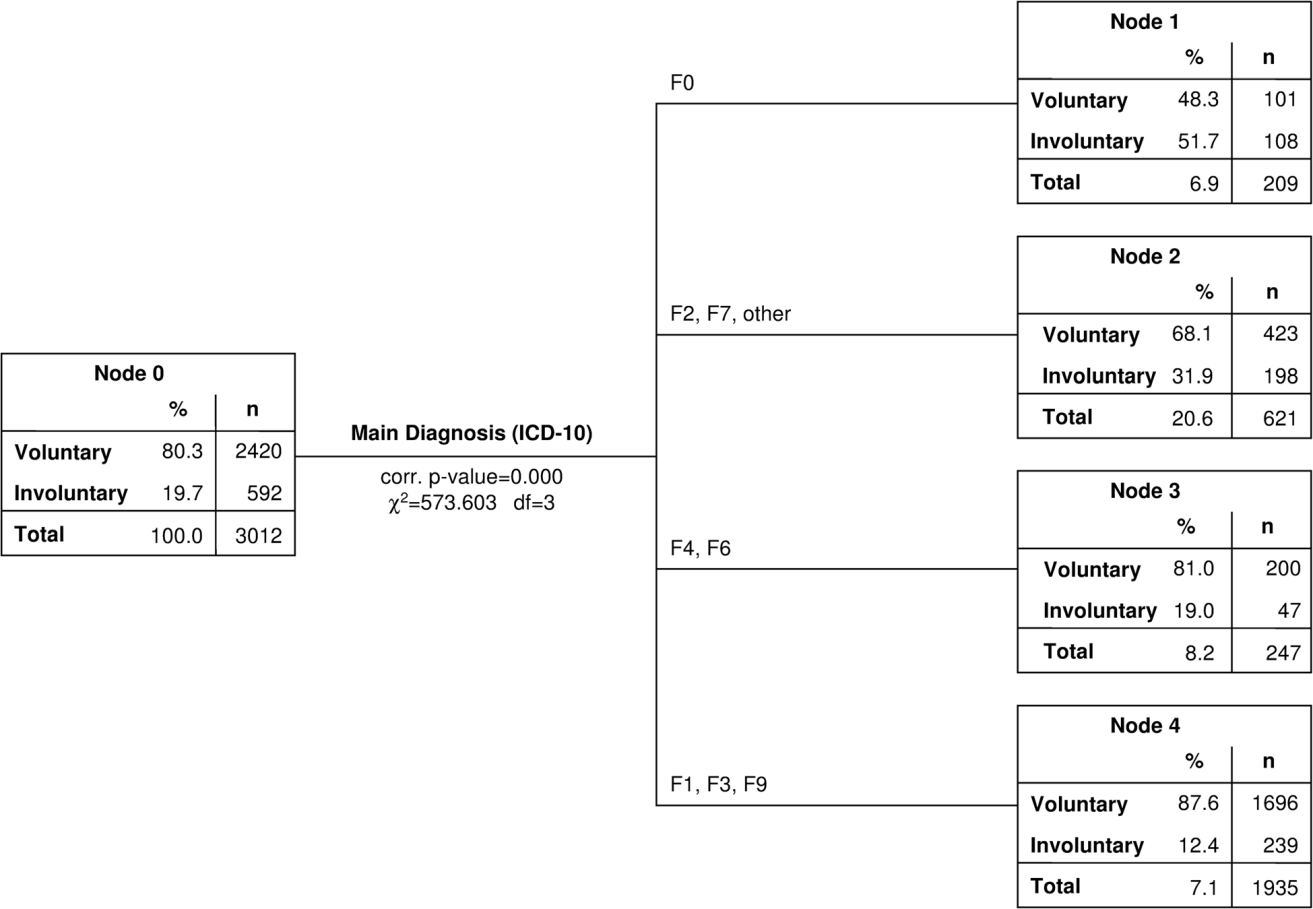
CHAID-model (test sample), 1st level: main diagnosis

The Exhaustive CHAID analysis yielded 16 statistically significant subgroups (terminal nodes). In the training sample, 93.1% of the involuntary group was predicted correctely, compared with 92.4% in the test sample. For both datasets, very similar areas under the curve (AUC) were found: Training AUC = 0.667; Test AUC = 0.665. These similarly correct classification rates indicate that the fitting process was performed adequately and satisfactorily, i.e. there is neither evidence for underfitting nor for overfitting. The main diagnosis was identified as the strongest predictor for involuntary treatment (χ^2^(3) = 573.603, corrected *p* < 0.001, first level of segmentation of the sample, see Fig. 1). Patients with organic mental disorders (ICD 10: F0) had the highest rate of involuntary admissions (51.7%); patients with psychotic disorders (F2) and patients with intellectual disabilities (F7) had the second highest rate (31.9%); patients with neurotic, stress-related and somatoform disorders (F4) and patients with personality disorders (F6) had a rate of involuntary admissions (19.0%) that was only slightly lower than the average rate of the entire study sample (19.7%). The group of patients with substance use disorders (F1) and affective disorders (F3) as well as the group of patients with behavioural and emotional disorders with onset in childhood or adolescence (F9) had the lowest rate of involuntary admissions (12.4%).

**Fig. 2:**
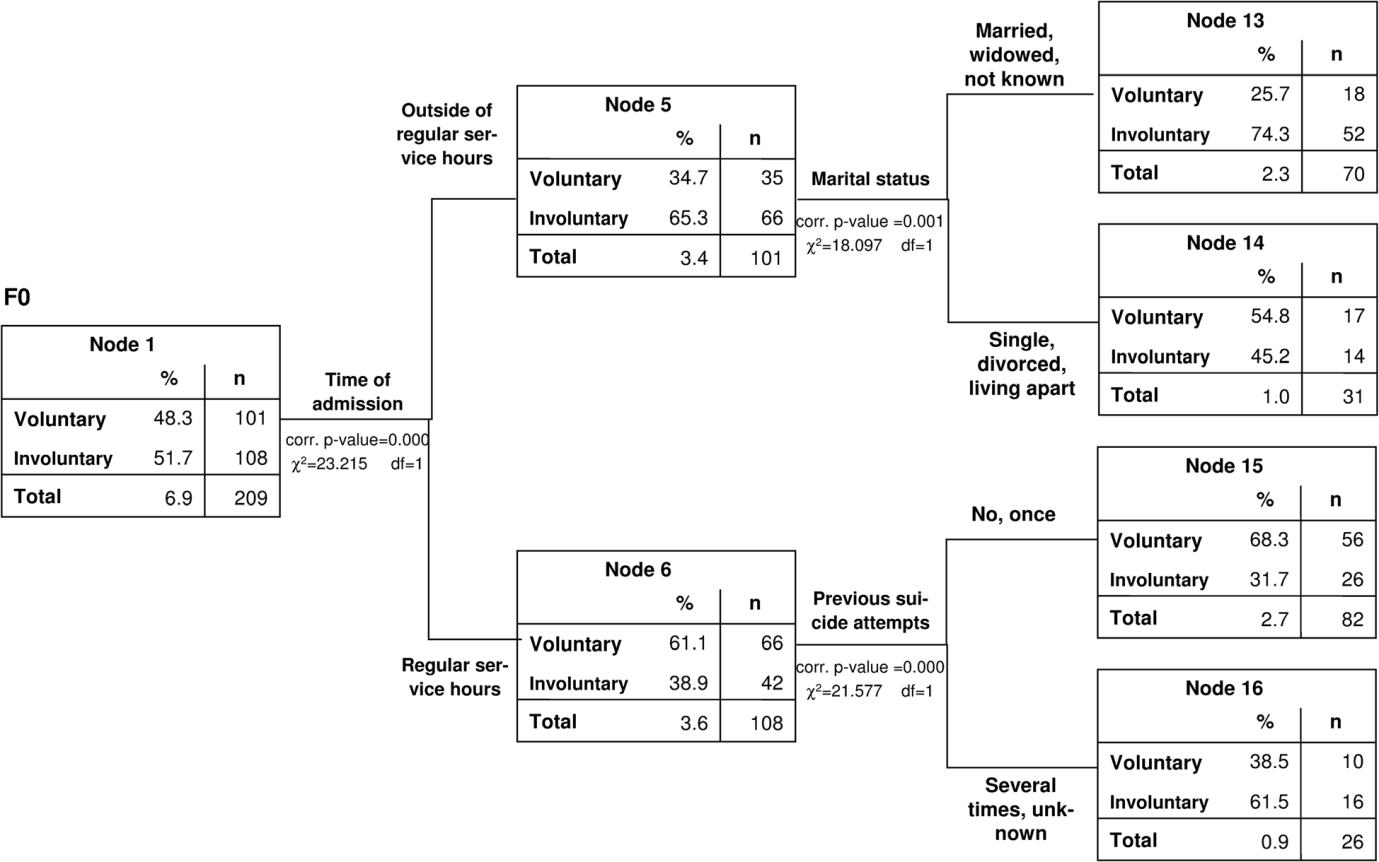
CHAID-Model (test sample), 2nd and 3rd levels: Group 1: organic mental disorders (mostly dementia) (ICD10: F0)

For the group of patients with organic mental disorders (ICD 10: F0), who were at the highest risk for involuntary treatment, admission outside of regular work hours (χ^2^(1) = 23.215, corrected *p* < 0.001), previous suicide attempts (χ^2^(1) = 21.577, corrected p < 0.001), and marital status (χ^2^(1) = 18.097, corrected *p* = 0.001), were identified as further critical variables (Fig. 2). Patients with organic mental disorders were more often subject to involuntary treatment if they were admitted outside regular working hours particularly if they were married or widowed (74.3% involuntary treatment in this highest risk group).

**Fig. 3:**
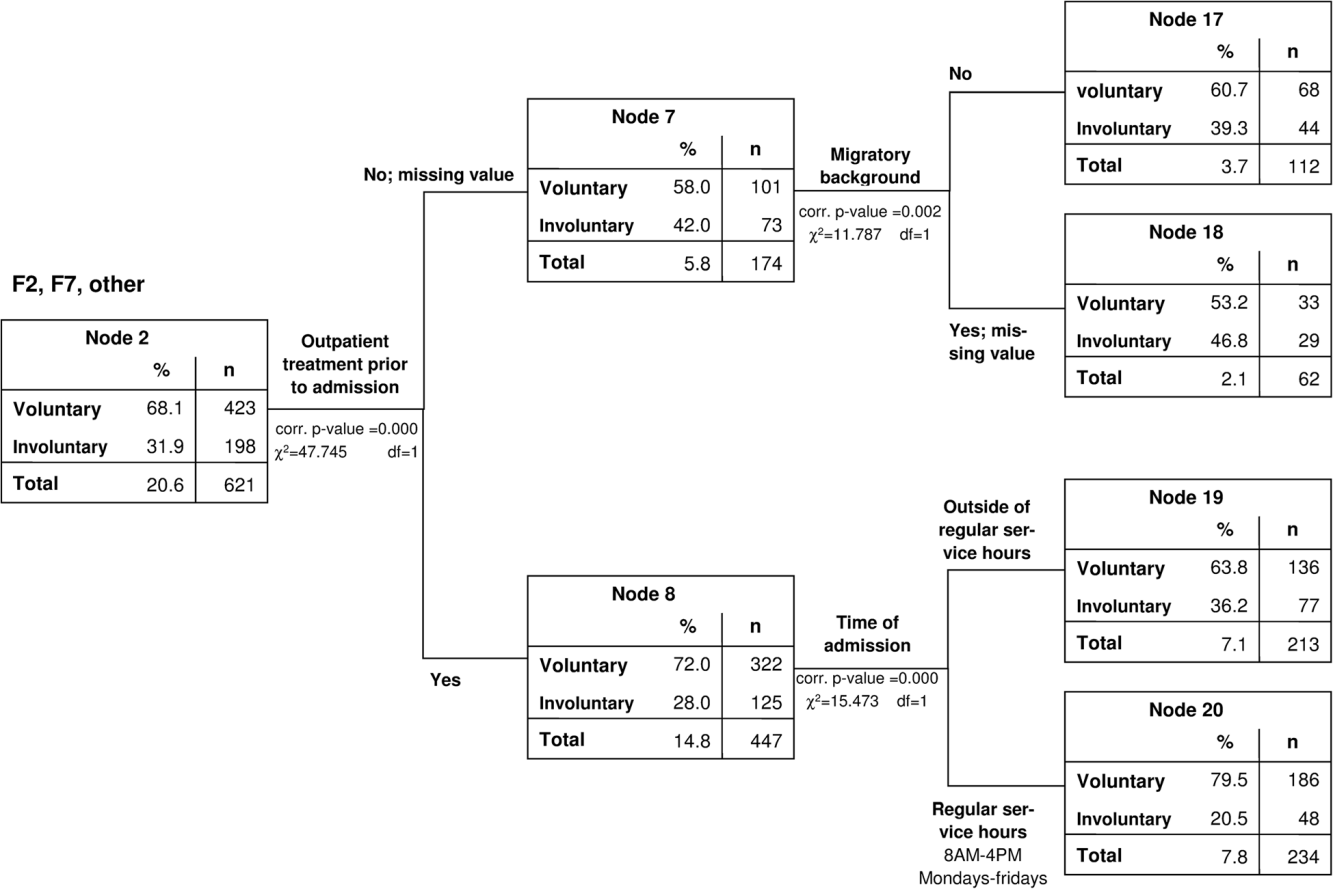
b) Group 2: psychotic disorders (ICD10: F2), intellectual disabilities (ICD10: F7), and other disorders (among them F5 and F8)

For the diagnostic groups with the second highest quota of involuntary treatment (ICD 10: F2 and F7, Fig. 3), lack of previous outpatient treatment (χ^2^(1) = 47.745 corrected *p* < 0.001), migratory background (χ^2^(1) = 11.787, corrected *p* = 0.002) and admission outside regular working hours (χ^2^(1) = 15.473, corredted *p* < 0.001) were found to be critical. Among patients who had had no regular outpatient psychiatric care, those with a migratory background were at highest risk (46.8% involuntary treatment). Patients with previous outpatient psychiatric care were more often admitted involuntarily (36.2% vs. 20.5%) if they attended an emergency service outside of regular working hours.

**Fig. 4:**
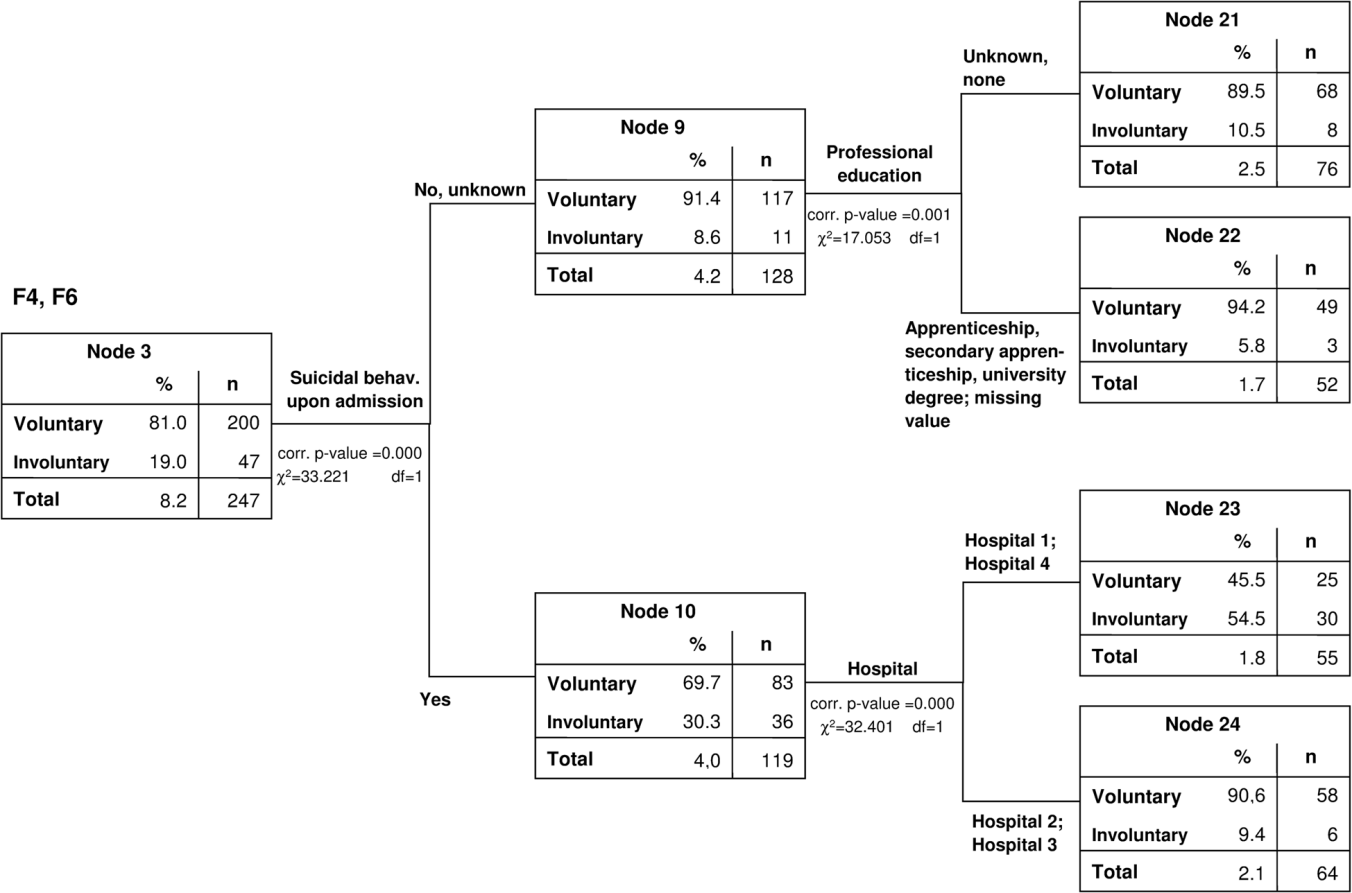
CHAID-model (test sample) 2nd and 3rd levels: Group 3: neurotic disorders (F4) and personality disorders (F6)

**Fig. 5:**
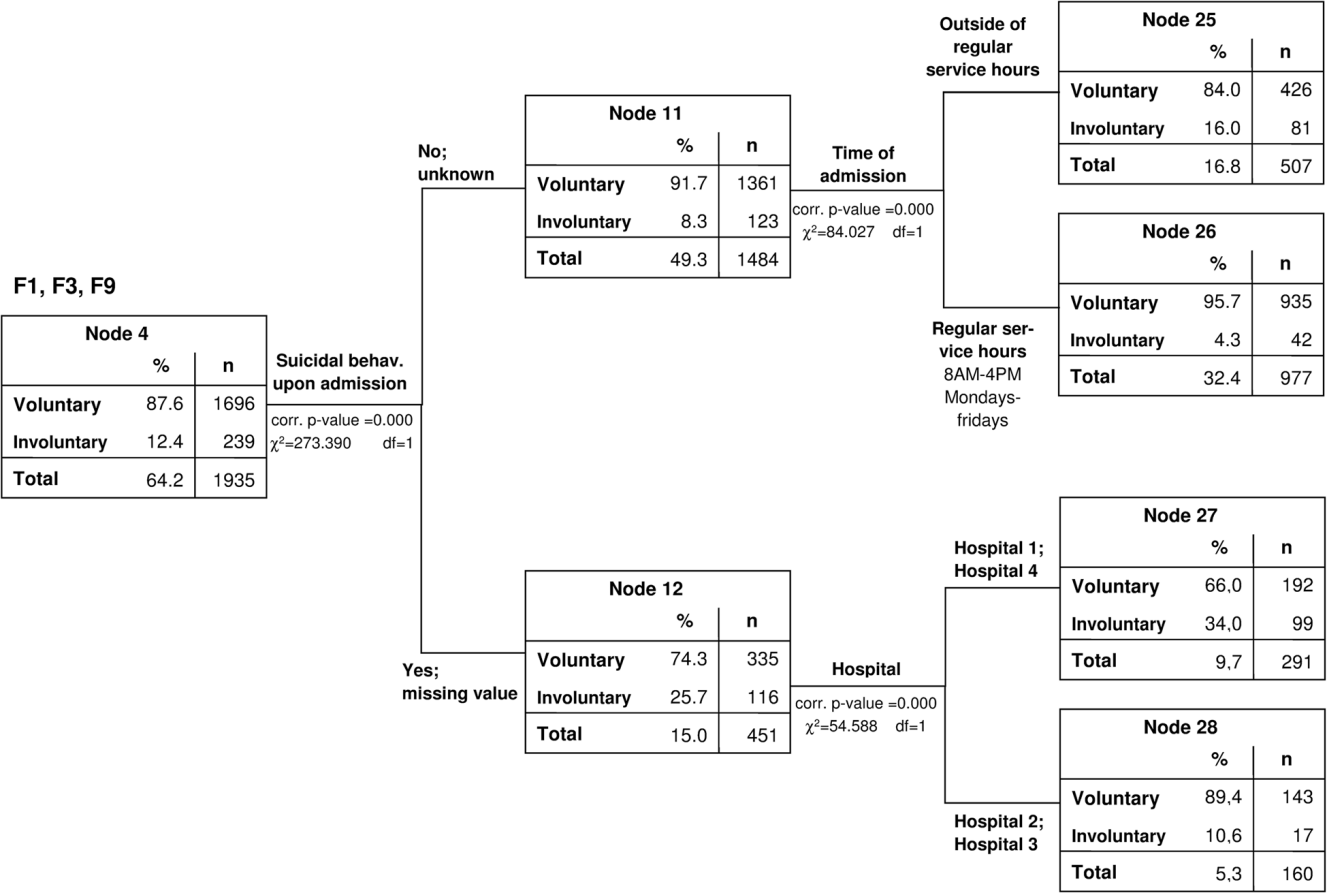
Group 4: affective disorders (F3), substance related disorders (F1) and behavioural and emotional disorders of childhood and adolescence (F9)

For the diagnostic groups with a quota of involuntary treatment around or below average (ICD 10: F4, F6, F1, F3, F9), suicidal and self-harm behaviour at admission were identified as most critical (F4, F6: χ^2^(1) = 33.221, corrected *p* < 0.001, Fig. 4, and F1, F3, F9: χ^2^(1) = 273.390, corrected p < 0.001, Fig. 5). In the next level of the CHAID decision tree, critical variables were admission out of regular working hours (χ^2^(1) = 84.027, corrected p < 0.001), the hospital in which the treatment took place (F4, F6: χ^2^(1) = 32.401, corrected p < 0.001; F1, F3, F9: χ^2^(1) = 54.588, corrected p < 0.001) and the patients’ level of professional training (χ^2^(1) = 17.053, corrected p < 0.001). When suicidal patients were admitted to either hospital 1 or 4, they were more likely to be treated under the German Mental Health Act (F4, F6: 54.5%; F1, F3, F9: 34.0%) compared to those admitted to hospitals 2 and 3 (F4, F6: 9.4%; F1, F3, F9: 10.6%). In search for an explanation for this finding, we carried out exploratory analyses and found that in the case of the two hospitals with higher quotas of involuntary treatment in suicidal cases, a higher proportion of Mental Health Act cases had been referrals from general hospitals (41.7 and 57.6% of the PsychKG cases in hospitals 1 and 4, compared to only 26.2 and 30.5% in hospitals 2 and 3 respectively). Non-suicidal patients with affective, substance related and behavioural and emotional disorders (F1, F3, F9) were more often admitted involuntarily (16.0% vs. 4.3%) if they attended an emergency service outside of regular working hours. Non-suicidal patients with neurotic and personality disorders (F4, F6) were at higher risk for treatment under the German Mental Health Act if they had no professional education (10.5% vs. 5.8%).

## Discussion

To identify risk factors for involuntary psychiatric treatment, we analysed the health records of 5764 cases treated in 2011 as inpatients in the four psychiatric hospitals of the metropolitan region of the City of Cologne in Germany.

We found persons with organic mental illness, intellectual disabilities, psychoses and a comorbidity of addiction and psychosis to be at increased risk for involuntary treatment under the North Rhine-Westphalia Mental Health Act (PsychKG NRW). These findings are in line with earlier studies from several countries. Among all clinical diagnoses, the most consistent and frequently reported association with detention has been for psychosis [3–6, 8–12, 22, 27, 28, 37] while some studies from Germany and Switzerland also reported associations with organic mental disorders and intellectual disabilities [4, 12]. The decision tree analysis identified the type of mental disorder as the strongest predictor for involuntary treatment. Among sociodemographic factors, older age, retirement (including early retirement), living in assisted accommodation, a migratory background and married or widowed status were found to be associated with an increased risk of involuntary treatment. Retirement, older age and widowed status most likely reflect both the advanced age of the majority of patients with organic mental disorders, particularly dementia, and the severity of mental disorders leading to early retirement. Due to the sociodemographic changes in central Europe with an aging population and the constant trend towards a higher life expectancy, elderly people with organic mental illness will continue to be of great interest. Our finding that this group of patients was frequently admitted involuntarily outside regular service hours suggests that this group may benefit from 24 h outreach psychiatric services and more intensive out-patient care to prevent involuntary admissions. Interestingly, high odds for detention of people with organic mental disorders were previously shown in studies from Germany and Switzerland [4, 12] but not from other countries. This discrepancy may be due to differences in the organisation of mental health care. For example, in the neighbouring country The Netherlands, elderly people with dementia are treated rather in specialised psychogeriatric nursing homes and not in general psychiatric hospitals. Hence, the large proportion of involuntarily admitted psychogeriatric patients in our study is probably not only due to the aging population, but also due to structural features of health care services. Currently, there is growing awareness of this issue and a trend towards establishing complementary health care structures.

Our finding of a migratory background as a risk factor for involuntary treatment is in line with many studies from different countries with various healthcare settings and ethnic groups [11, 13, 19–21, 26, 28, 38–41]. This may reflect insufficient integration of migrants, leading to poor use of out-patient and psychosocial services mostly due to cultural and language barriers [42], or low trust of this patient group in receiving help from services based on previous experiences of social exclusion. These problems are probably associated with increased stress levels and inappropriate or delayed service use, factors which may well contribute to acute involuntary admissions. According to the decision tree analysis, the role of migratory background was most critical for the group of patients with psychosis (ICD10: F2) and particularly for those without regular out-patient treatment. It seems plausible that high stress levels, communication problems due to cultural and language barriers and the various aspects of experiencing oneself as an outsider will interact with and worsen the effects of poor insight and low treatment adherence in a subgroup of people with psychosis. This interpretation is in line with an intercorrelation of migratory background and schizophrenia as risk factors for detention shown in a deprived area of Dublin, Ireland [22]. These findings point out the need for more intense measures to promote the integration of migrants into society and the necessity to establish crisis services for this specifically vulnerable group, including mother-tongue services.

In our study, patients with substance-related and affective disorders had a relatively low risk of being treated compulsorily. In line with our finding, a recent study from Greece reported that a diagnosis of an affective disorder, especially unipolar depression, yielded a protective effect against involuntary hospitalization [13]. Only few studies looked differentially at the subgroups of affective disorders, and - not surprisingly - these reported that a diagnosis of bipolar disorder was associated with a high risk for detention [7, 16]. Regarding substance use disorders, reports from the literature are somewhat contradictory with two studies from the Netherlands and Norway reporting high risks for detention [21, 23].

Among the mental disorders with average or low risks of involuntary treatment (substance related, affective, neurotic, personality and behavioural and emotional disorders), the decision tree analysis identified suicidal behaviour and self-harm as the strongest predictors. It is noteworthy that the risk of involuntary treatment of patients with suicidal and self-harm behaviour was higher in two of the four psychiatric hospitals. Interestingly, we found a higher proportion of Mental Health Act referrals from emergency units of general hospitals in those hospitals. Although we cannot exclude the possibility that the psychiatric hospitals themselves dealt with suicidal patients in different ways, it appears more plausible that the interaction in the emergency room was critical. Assigning a key role to preventing involuntary psychiatric admissions to the referring general hospitals may be promising. Efforts to increase the training of emergency room staff members in deescalation techniques and dealing with suicidality are warranted. Also, introducing psychiatric consultation services in general hospitals may be warranted. We conclude that interventions aimed at reducing involuntary psychiatric admissions will need to include general hospitals.

For the large groups of non-suicidal patients with affective and substance-related disorders, the risk of treatment under the German Mental Health Act increased if admission took place outside of regular service hours. The same was true for patients with psychotic disorders or intellectual disabilities in regular out-patient treatment. This finding is in line with previous studies from Germany and Norway [11, 12, 23]. It seems plausible that individuals attending the emergency services outside of regular service hours will be more severely ill than other patients and therefore they will be subject to involuntary treatment more often. This may partly explain the association between admission outside of regular service hours and involuntary treatment. However, this association may also hint at deficits in the organization of psychiatric emergency services such as low staffing levels at night and weekends. As both explanations are plausible, their relative merit cannot be determined based on data available from this study.

### Strengths and limitations

A major strength of our study is the detailed in-depth analysis of health records of a large sample of psychiatric in-patients representative for a complete metropolitan region of Germany. In addition to administrative and routinely available data, we screened all available data sources for a detailed list of sociodemographic and clinical items previously shown to be associated with detentions. We included all cases treated under the Mental Health Act in the City of Cologne over the course of one year (except from a defined small part of the northern city) and we also included a large sample of voluntary patients from the same hospitals and year who served as controls. We obtained data from all four psychiatric hospitals which serve this region and admit patients under the Mental Health Act, thus avoiding sampling bias and ensuring the generalizability of our findings. The City of Cologne comprises a wide range of sociodemographic neighbourhoods. Community-based social psychiatric services are organized in similar ways all over the region and a single Municipal Court is responsible for all detentions in the city. Hence, variation due to systemic factors such as differing jurisdictions and/or major differences in out-patient services was minimized.

In addition, our statistical analysis went beyond previous studies through the application of the CHAID decision tree analysis. For the purpose of our research questions, CHAID was superior to logistic regression [43] as it allowed us to identify interactions between different risk factors for detention and develop a classification of risks leading to tailored suggestions for preventive interventions. By using a CHAID algorithm with a maximum depth of three levels we developed a model which is easily understood by clinicians. The resulting model appears to be adequately accurate with no overfitting. Further improvements of the model fit may be accomplished by ensemble methods such as bagging, boosting or stacking, which we intend to use in a future project employing machine learning algorithms.

The shortcomings of our study are pertinent to its retrospective design. We collected clinical and administrative data from existing medical records and these data were incomplete for research purposes. Hence, we have no information on potentially important features that may drive involuntary hospitalization, such as symptom severity, level of psychosocial functioning, insight or perceived social support. Lack of this information prevents the development of a comprehensive risk model for involuntary psychiatric hospitalizations. Moreover, the retrospective nature of the study does not allow for making causal interpretations, as the observed relations may be influenced by unobserved third variables. In addition, although we searched all available data sources in-depth, the number of missing values was considerable for some sociodemographic variables. This may have reduced the power of our study to detect possible links between sociodemographic factors and the legal status of in-patient treatment. Finally, although there are many similarities between our findings and results from another recent study from Germany [11, 12] as well as several studies from metropolitan regions of other European countries [7, 9, 13, 16, 26, 44], we cannot be sure how far the generalizability of our findings goes. Differences between health systems across different countries and differences in availability and quality of health services depending on the degree of urbanicity may well lead to increased or decreased detention rates. Hence, results may be very different in other countries and in rural regions.

### Conclusions

Patients with organic mental disorders had the highest likelihood of involuntary treatment. Support measures, e.g. specific training of relatives and professionals providing home care, may help manage crises in at-home situations and avoid hospital admissions, in particular at night or during weekends [45]. Measures for the prevention and management of mental crises in persons with intellectual disabilities should be established in a similar way [46].

There is also a great need for action in improving mental healthcare for migrants. Local networks of mental health care providers need to become fully accessible and offer linguistically and culturally appropriate services. Measures to be implemented may include training of staff in cultural sensitivity and the use of interpreters in diagnostic and treatment procedures [47].

We cannot be sure about the nature of the association between the time of admission in a psychiatric hospital and the legal status. It does seem plausible, however, that emergency hospital services with lower staffing levels may increase the risk of involuntary admission. In this case, community-based, outreach crisis intervention services may lower the need for hospital attendance and this may lower involuntary admission rates [48, 49]. Finally, it may be warranted to improve deescalation skills in general hospital emergency departments and other emergency services. Deescalation training of emergency professionals may be crucial in reducing involuntary psychiatric in-patient admissions. These factors appear to be not specific to the situation in Cologne or Germany.

In summary, a variety of interventions may help to improve health services and reduce involuntary treatment in different risk groups for involuntary admissions by addressing modifiable risk factors as identified in our analysis. Currently, only limited evidence is available for the efficacy of preventive measures targeted at certain risk groups, pointing to a need to develop, implement and evaluate such programs [48].

## Abbreviations

BGB: German Civil Code, §§ 1896 ff. BGB (Legal Guardianship)
CHAID: Chi-squared automatic interaction detection
DGPPN: German Association for Psychiatry, Psychotherapy and Psychosomatics
M: Mean
NRW: North Rhine-Westphalia
PsychKG NRW: North Rhine-Westphalia Mental Health Act
SD: Standard deviation
